# Transcriptome profiling of hypothalamus-pituitary-ovary axis provides insights into egg-laying interval differences between european meat pigeons and shiqi pigeons

**DOI:** 10.3389/fgene.2025.1676255

**Published:** 2025-10-16

**Authors:** Xinlei Wang, Liyu Yang, Ruiting Li, Liheng Zhang, Zhanbing Han, Runzhi Wang, Qiang Li, Dingding Zhang, Mingjun Yang, Pengkun Yang

**Affiliations:** ^1^ College of Animal Science and Technology, Henan University of Animal Husbandry and Economy, Zhengzhou, China; ^2^ Nanjing Institute of Animal Husbandry and Poultry Science, Nanjing, China; ^3^ Henan Tiancheng Pigeon Industry Co., Ltd, Pingdingshan, China

**Keywords:** european meat pigeons, shiqi pigeons, hypothalamic-pituitary-ovarian axis, reproduction, egg-laying interval

## Abstract

**Introduction:**

The long egg-laying interval of pigeons leads to low egg production, and the hypothalamus-pituitary-ovary (HPO) axis plays a crucial role in regulating the egg-laying process of poultry. European meat pigeons have a shorter egg-laying interval than Shiqi pigeons, but the molecular mechanism underlying this difference remains unclear.

**Methods:**

Reproductive phenotypic data of 300 pairs of pigeons from each breed were collected for 6 months. Five 2-2.5-year-old female pigeons from each breed were selected, and their hypothalamus, pituitary, and ovary tissues were collected for transcriptome sequencing. Differentially Expressed Genes (DEGs) were identified, and Gene Ontology (GO) and Kyoto Encyclopedia of Genes and Genomes (KEGG) enrichment analyses were performed.

**Results:**

The egg-laying interval of European meat pigeons (32.76 ± 3.25 days) was significantly shorter than that of Shiqi pigeons (33.11 ± 3.86 days, P=0.024). A total of 39, 101, and 199 DEGs were identified in the comparisons of SH vs EH, SP vs EP, and SO vs EO, respectively. DEGs in the hypothalamus and pituitary were enriched in pathways such as thyroid hormone transport and calcium-mediated signaling; DEGs in the ovary were enriched in pathways such as embryonic development and steroid biosynthesis. The thyroid hormone signaling pathway (in the hypothalamus and pituitary) and the steroid hormone biosynthesis pathway (in the ovary) were significantly enriched, and key genes such as StAR, EYA1, HAND2, HOXB8, and NRN1 were identified.

**Discussion:**

The hypothalamus-pituitary-ovary axis regulates the egg-laying interval of pigeons through tissue-specific pathways. Among them, the thyroid hormone signaling pathway controls upstream hormone secretion, and the steroid biosynthesis pathway affects follicle maturation. The identified key genes may serve as targets for shortening the egg-laying interval.

## 1 Introduction

As a high-quality product with high nutrition and high protein, pigeons are highly favored by consumers. The ancient Chinese medical masterpiece “Compendium of Materia Medica” records that “one pigeon is better than nine chickens”, which fully shows that pigeons are ingredients with extremely high nutritional value (Long and Ye, 2011), and its high-quality nutritional content is significantly higher than that of other poultry. Pigeon meat is rich in mineral elements and vitamins, and also possesses medicinal properties. Moreover, the meat is tender and smooth, easy to digest and absorb, and is an ideal tonic food for humans (Long and Ye, 2011). Compared with other poultry, pigeons have unique reproductive characteristics such as monogamy, induced ovulation, and parent pigeons feeding squabs. Pigeons lay only two eggs per egg-laying cycle, with a 48-hour interval between the two eggs (Nepote, 1999). For natural incubation and feeding, the interval between two egg-laying periods is 30–40 days. Breeding pigeons can lay only about 20–28 eggs annually and produce 16 to 22 squabs annually. After laying two eggs, pigeons enter the incubation state.

Egg-laying interval is one of the important indexes to measure the egg-laying-related traits of pigeons, and there are significant differences in egg-laying interval among different breeds of pigeons. European meat pigeons have excellent reproductive performance and extremely high productivity. The average egg-laying interval is about 31 days. They also have excellent reproductive performance such as strong brooding ability (Tang and Mu, 2018). Shiqi pigeons are produced in Shiqi area, Zhongshan County, Guangdong Province, China. They are pigeons used for both meat and ornamental purposes and have a history of more than a hundred years. Shiqi pigeons have characteristics such as gentle temperament, strong adaptability, tolerance to coarse feed and good nesting behavior. The average egg-laying interval is around 32 days (Chunyu et al., 2018). To guarantee the continuity of egg production, the development of follicles in poultry is hierarchical. Nevertheless, in contrast to chickens, pigeons merely lay two eggs during the laying period and have a considerable interval between laying eggs. Follicle development is a complex biological process, which is precisely regulated by reproductive hormones and related genes.

The reproduction of poultry is genetically dependent on the regulation of a series of reproductive hormones in the hypothalamic-pituitary-gonadal axis (HPG). The hypothalamus-pituitary-ovarian axis (HPO) controls the reproductive process of hens and influences follicular selection, development, atresia, and ovulation (Shin-ichi, 1986; Zhao et al., 2023). The egg-laying process of chickens involves a series of hormonal changes coordinated by the HPO axis (Xu et al., 2023). The preoptic and arcuate nucleus neurons of the hypothalamus can secrete gonadotropin-inhibiting hormone (GnIH) and gonadotropin-releasing hormone (GnRH). These are a pair of extremely important regulatory neuropeptides in the hypothalamus and play a key regulatory role in the hormonal regulation of the HPG in poultry. The central nervous system analyzes and integrates various information from external stimuli and self-feedback. After the hypothalamus receives this information, GnRH is released in a pulsatile manner to stimulate the anterior pituitary to release follicle-stimulating hormone (FSH) and luteinizing hormone (LH). FSH and LH mainly act on the ovary to promote follicular maturation, and secrete estrogen (E2) and progesterone (P), thereby maintaining the egg-laying state of hens (Oduwole et al., 2021; Prastiya et al., 2022). In addition, the hypothalamus and pituitary gland also secrete other hormones and neuropeptides that are involved in the regulation of avian ovarian steroid hormone synthesis, follicular development and ovulation through HPG, such as growth hormone (GH), oxytocin and prolactin (Shin-ichi, 1986; Hrabia et al., 2011; Hrabia, 2015; Hu and Zadworny, 2017). Prolactin PRL promotes commitment to parental pigeon care of offspring without simultaneously inhibiting reproductive function or HPG axis activity (Farrar et al., 2021). The expression changes of GnIH and GnRH genes in the hypothalamus of pigeons during different stages of reproduction confirm that the expression of GnIH and GnRH genes is related to the transition of the female pigeon to different reproductive stages (Zhang Rui et al., 2018). Integrated analysis of transcriptome sequencing analyses of multiple tissues from high and low laying Goodyear chickens, as well as *in vivo* tissue-specific overexpression assays, demonstrated that liver- and ventral lipid-specific endocrine factors target the HPO axis to regulate chicken egg production (Wang et al., 2024).

The present study aimed to conduct a comparative transcriptomic analysis of the hypothalamus-pituitary-ovary axis in European meat pigeons and Shiqi pigeons during the egg-laying interval through RNA sequencing, with the goal of identifying candidate genes and signaling pathways that might be involved in the regulation of egg-laying interval, thereby laying a data foundation for shortening the egg-laying interval of pigeons.

## 2 Material methods

### 2.1 Animals

This experiment took European meat pigeons and Shiqi pigeons (meat pigeon data and samples provided by Henan Tiancheng Pigeon Industry Co., Ltd.) as the research objects. For each breed, 300 pairs of pigeons were selected for a 6-month statistical analysis of reproductive phenotypic data, including the number of eggs laid, the number of fertilized eggs, egg-laying intervals, and hatching rates. Afterwards, five female European meat pigeons and Shiqi pigeons aged 2–2.5 years were randomly selected for slaughter. Hypothalamus, pituitary, and ovarian tissues were collected, immediately frozen in liquid nitrogen (−196 °C), and then stored in a refrigerator at −80 °C for future use. All experiments in this study were conducted in accordance with a protocol approved by the Institutional Animal Care and Use Committee (IACUC) in China, under ethical approval code HNUAHEER 2425106.

### 2.2 RNA extraction, library construction, and sequencing

In accordance with the manufacturer’s guidelines, total RNA was extracted utilizing the Trizol Reagent Kit (Invitrogen, Carlsbad, CA, United States). The quality of the extracted total RNA was evaluated using an Agilent 2100 Bioanalyzer (Agilent Technologies, Palo Alto, CA, United States) and further verified through agarose gel electrophoresis without RNase contamination. Subsequently, rRNA was removed. For library preparation, the BGI Optimal Series Dual Module mRNA Library Construction Kit was employed. Initially, mRNA was isolated via denaturation treatment followed by enrichment using oligo (dT) magnetic beads. Fragmentation of mRNA resulted in smaller fragments after treatment with a fragmentation reagent. The first strand cDNA synthesis was conducted through reverse transcription employing random hexamer primers derived from these mRNA fragments. Following this step, second strand cDNA synthesis occurred. Next, the 3′ends of the cDNA were repaired and an A base added before ligating adapters to them. PCR amplification ensued after denaturing the PCR products into single strands. Linear DNA that remained uncyclized underwent digestion to yield a single-stranded circular library. Ultimately, phi29 amplification generated DNA nanoballs (DNB), which were sequenced on BGI’s sequencing platform utilizing high-density DNA nanopore technology alongside cPAS and PE100/PE150 methodologies.

### 2.3 Bioinformatics analysis

Raw sequencing data from the hypothalamic-pituitary-ovarian (HPO) axis tissues of European meat pigeons (EH, EP, EO) and Shiqi pigeons (SH, SP, SO) were quality-filtered using SOAPnuke (v1.5.6) to obtain clean reads. Subsequently, the Dr. Tom multi-omics data mining system (https://biosys.bgi.com) were used for data analysis, plotting and mining. For differential gene analysis. In order to further explore the related gene functions in depth, we perform Gene Ontology (GO) (http://www.geneontology.org/) and Kyoto Encyclopedia of Genes and Genomes (KEGG) (https://www.kegg.jp/) enrichment analysis on differential genes using Phyper based on hypergeometric test. Taking *P-*value <0.05 as the threshold, those meeting this condition are defined as significantly enriched in candidate genes.

### 2.4 Data processing

Independent samples t-test was used to compare the egg-laying intervals among different breeds. Statistical analysis was performed using SPSS 27.0 software, and the data were presented as mean ± standard deviation (Mean ± SD).

## 3 Result

### 3.1 Reproductive phenotypic statistics

A 6-month statistical analysis of 300 pairs of European meat pigeons and 300 pairs of Shiqi pigeons showed differences in reproductive phenotypic indicators (Table 1). Among the detected indicators, only the egg-laying interval exhibited a significant difference between the two breeds. No significant differences were observed in the number of eggs laid, number of fertilized eggs, or hatching rates (*P-*value >0.05).

**TABLE 1:** Comparison of reproductive performance among different pigeon breeds.

Breed	Egg-laying interval	Number of eggs laid (6 months)	Number of fertile eggs (6 months)	Fertility rate (%)	Hatching rate (%)
European meat pigeons	32.76 ± 3.25^a^	12.00 ± 1.52	10.71 ± 1.28	90.23 ± 0.11	87 ± 0.13
Shiqi pigeons	33.11 ± 3.86^b^	12.01 ± 1.53	10.80 ± 1.34	90.87 ± 0.12	89 ± 0.13

The absence of the same capital letter after the data in the same column indicates a significant difference between groups (*P*-value <0.05).

### 3.2 Differential expression analysis

**FIGURE 1:**
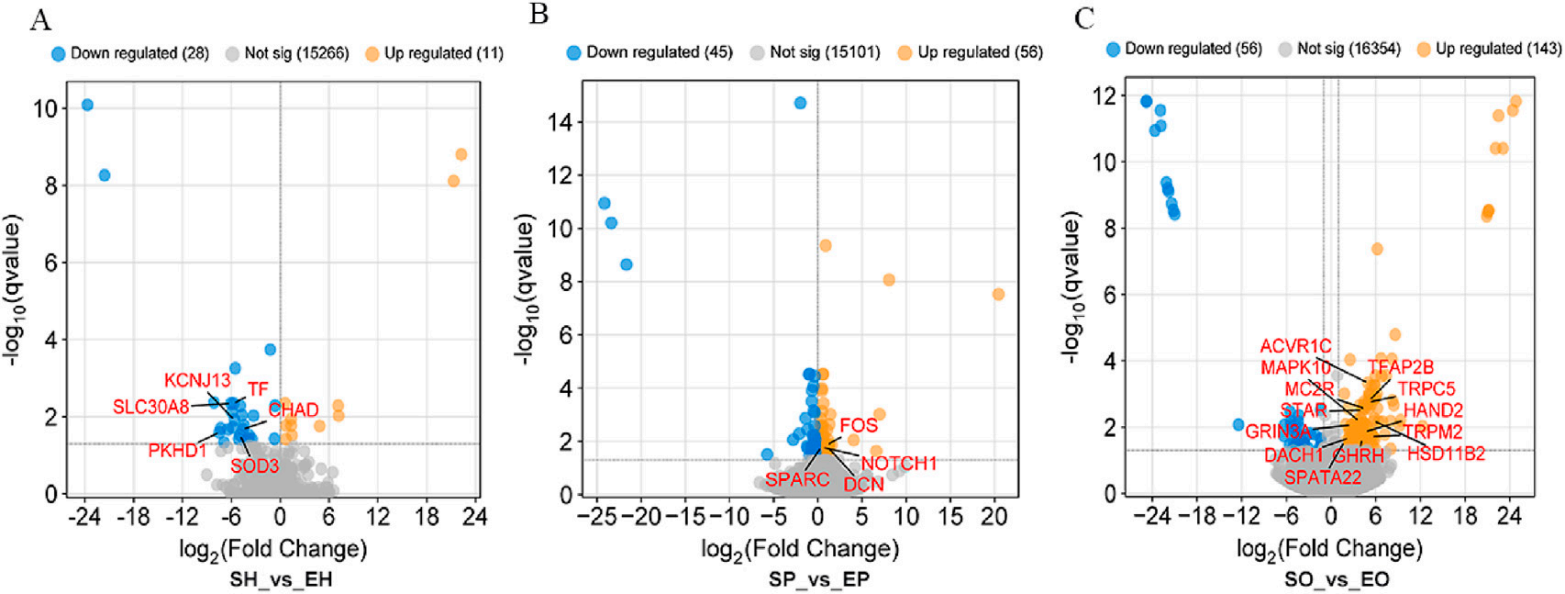
Differential gene expression profiles in **(A)** hypothalamic, **(B)** pituitary, and **(C)** ovarian tissues. Red/blue dots represent up-/downregulated genes (|log2FC| ≥ 2, FDR <0.05).

The hypothalamus, pituitary gland, and ovarian tissues of Shiqi pigeons and European meat pigeons were analyzed using transcriptome sequencing technology. A total of 191.4 Gb clean reads were obtained from 30 samples after data filtering, which involved removing low-quality reads (Q-value <20), reads containing adaptor sequences, and reads with ambiguous bases (N content >5%). The average clean data per sample was 6.38 Gb, with an average mapping rate of 77.33% to the reference genome. The Q20/Q30 base ratio: For all samples, the Q20 was 98.5%–98.77%, and the Q30 was 94.06%–95.14% (Supplementary Tables 1, 2). In the comparisons of SH vs EH, SP vs EP and SO vs EO; 39 (11 upregulated and 28 downregulated), 101 (56 upregulated and 45 downregulated), and 199 (143 upregulated and 56 downregulated) differentially expressed genes (DEGs) were identified respectively, with FDR <0.05 and |log2 (fold change)| ≥ 2 (Figure 1A). Overall, there were significant differences in gene expression in HPO axis tissues between the two pigeon species, with the ovary tissue having the highest number of DEGs (199), suggesting that the ovary is a key tissue in the regulation of egg-laying interval.

### 3.3 Differently expressed gene function analysis

**FIGURE 2:**
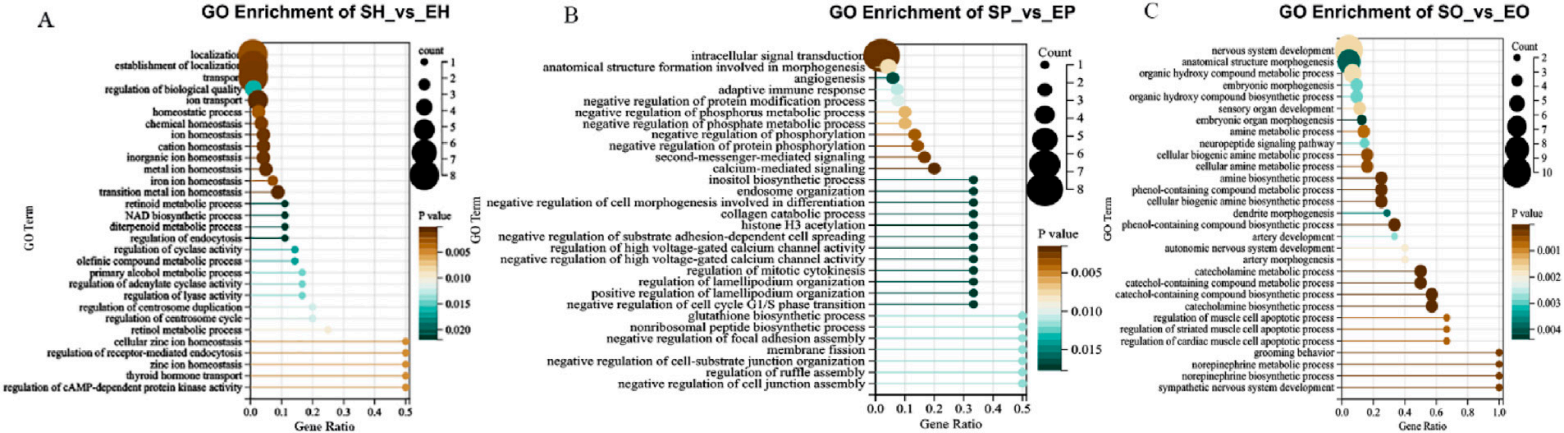
Biological process terms accounted for the highest proportion in the top 30 GO terms. **(A)** Top GO terms for source genes of DEGs of SH vs EH; **(B)** top GO terms for source genes of DEGs of SP vs EP; **(C)** top GO terms for source genes of DEGs of SO vs EO.

Enrichment analysis provides the most detailed GO term information, which can facilitate in-depth analysis of the regulation mechanism of HPG axis on egg-laying intervals in different breeds of pigeons. We focused on the biological process (BP) and selected the top 30 pathways to focus on. In SH vs EH group, the DEGs are mainly related to transition metal ion homeostasis, ion transport, metal ion homeostasis. In addition, we noticed that the pathways related to reproduction regulation, such as regulation of receptor-mediated endocytosis, thyroid hormone transport, regulation of receptor - mediated endocytosis, regulation of centrosome duplication, regulation of cyclase activity, retinol/retinoid metabolic process (Figure 2A). In SP vs EP group, DEGs are mainly assigned to calcium-mediated signaling, intracellular signal transduction, second-messenger-mediated signaling, cell - cycle regulation, cell Signaling and cell Morphogenesis and Adhesion (Figure 2B). In SO vs EO group, most DEGs are concerned with sensory organ development, nervous system development, embryonic morphogenesis, embryonic organ morphogenesis, steroid biosynthetic process, system development (Figure 2C).

### 3.4 KEGG pathway enrichment analysis

**FIGURE 3:**
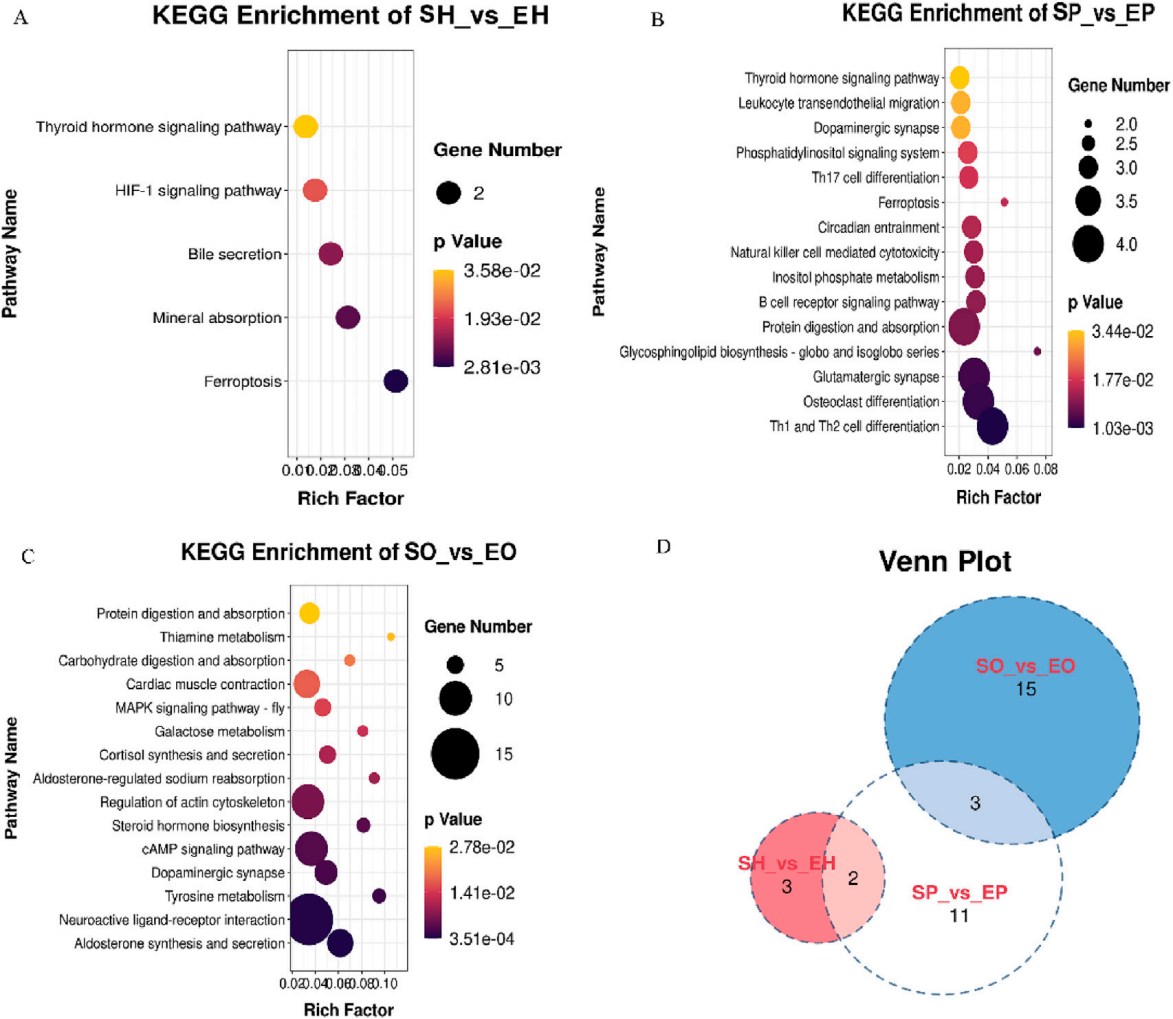
The KEGG pathway enriched some important pathways closely related to reproduction. **(A)** KEGG pathways for source genes of DEGs of SH vs EH; **(B)** KEGG pathways for source genes of DEGs of SP vs EP; **(C)** KEGG pathways for source genes of DEGs of SO vs EO; **(D)** Venn diagram showing shared and unique KEGG pathways among comparative groups: Hypothalamus (SH vs EH), Pituitary (SP vs EP), and Ovary (SO vs EO) in Shiqi and European pigeons.

KEGG pathway enrichment analysis of differential genes in HPG axis-related tissues. The DEGs identified SH vs EH group were mainly enriched in the ferroptosis, mineral absorption, Bile secretion, HIF-1 signaling pathway, glycosphingolipid biosynthesis-globo and isoglobo series, Neuroactive ligand-receptor interaction. In the pituitary, the DEGs mainly enriched in the Th1 and Th2 cell differentiation, osteoclast differentiation, glutamatergic synapse, inositol phosphate metabolism, glycosphingolipid biosynthesis-globo and isoglobo series, ferroptosis pathways are significantly enriched. Notably, the thyroid hormone signaling pathway was enriched in both hypothalamic and pituitary tissues,a pathway previously reported to be involved in reproductive regulation (Figure 3D). In the ovary, neuroactive ligand-receptor interaction, aldosterone synthesis and secretion, steroid hormone biosynthesis, tyrosine metabolism, cAMP signaling pathway, dopaminergic synapse, regulation of actin cytoskeleton, cortisol synthesis and secretion, aldosterone-regulated sodium absorption.

### 3.5 Gene expression cluster analysis

**FIGURE 4:**
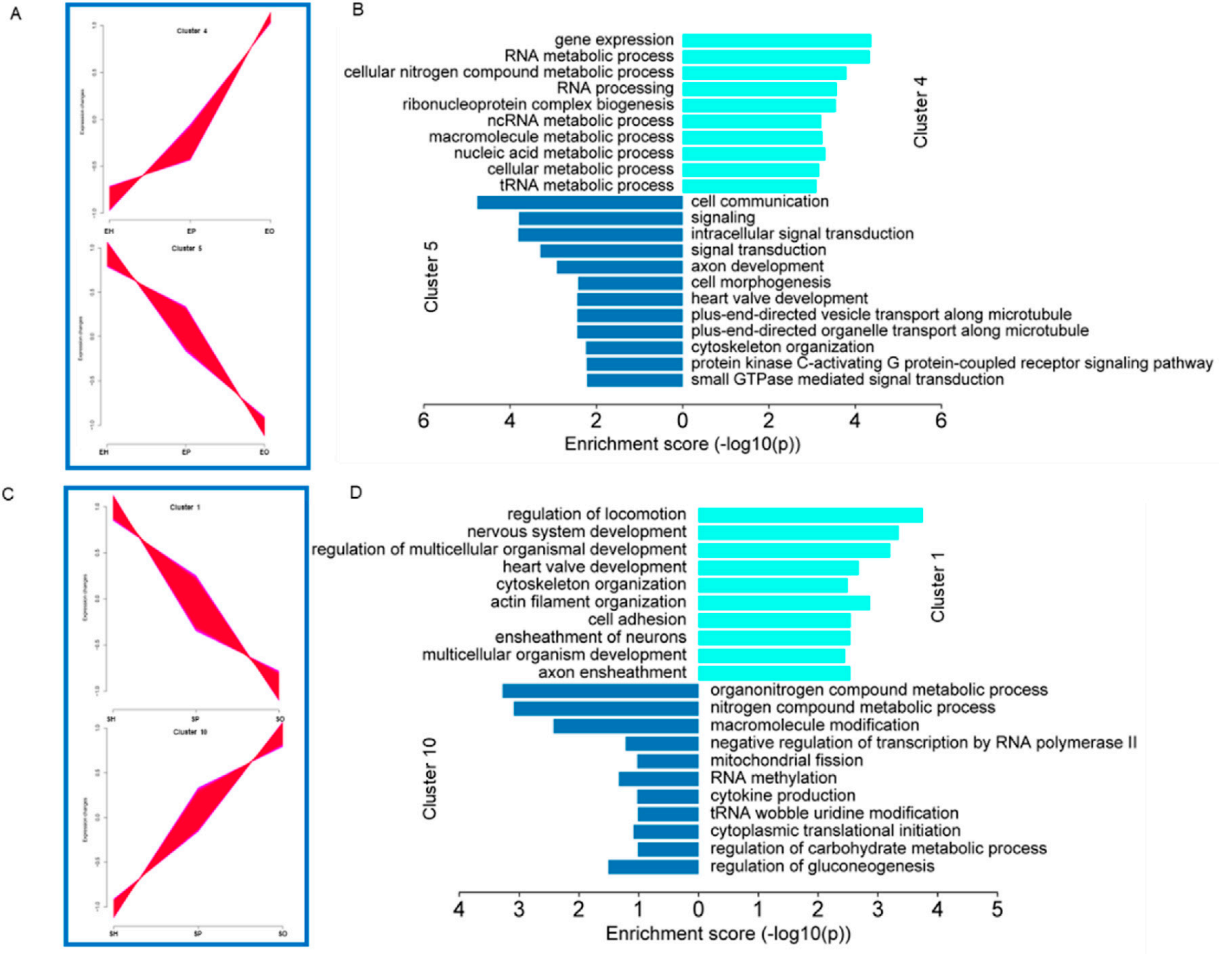
Gene cluster expression and function analysis. **(A-C)** Cluster analysis of all genes based on expression. All the genes clustered into ten clusters, clusters with opposite expression trends were classified into one group. **(B-D)** Analysis of the functions of genes in the different groups. The bar plot shows significant GO terms by gradient legend as *P*-value <0.05.

Expression pattern analysis was used to classify the gene expression change trends of genes expressed in the HPO axis of different pigeon breeds, and to infer the possible relationships and specific functions of expressed genes among different tissues. The genes expressed in the HPO axis of Shiqi pigeons and European meat pigeons were clustered into a total of 10 clusters. According to their dynamic expression changes, we selected two groups with opposite expression trends for analysis. In European meat pigeons, cluster 4 and cluster 5 have opposite expression trends (Figure 4A). GO function enrichment analysis shows that the genes in cluster 4 are mainly related to functions such as gene expression, RNA metabolic process, and cellular nitrogen compound metabolic process; the genes in cluster 5 are mainly related to cell communication, signaling, intracellular signal transduction, signal transduction, axon development, cell morphogenesis (Figure 4B). In Shiqi pigeons, cluster 1 and cluster 10 have opposite expression trends (Figure 4C). The GO function enrichment analysis of cluster 1 genes shows that it is mainly related to functions such as regulation of locomotion, nervous system development, and regulation of multicellular organismal development. The GO function enrichment analysis of cluster 10 genes shows that it is mainly related to functions such as organonitrogen compound metabolic process, nitrogen compound metabolic process, and macromolecule modification. In summary, we found that there is some similarity between the genes expressed in European meat pigeon cluster 5 and Shiqi pigeon cluster 1, which are mostly related to the growth and development of cells or tissues, nervous system development, heart valve development, positive regulation of cell growth, positive regulation of developmental growth, positive regulation of cell population proliferation (Figures 4B,D).

### 3.6 Pathway-gene interaction network analysis

**FIGURE 5:**
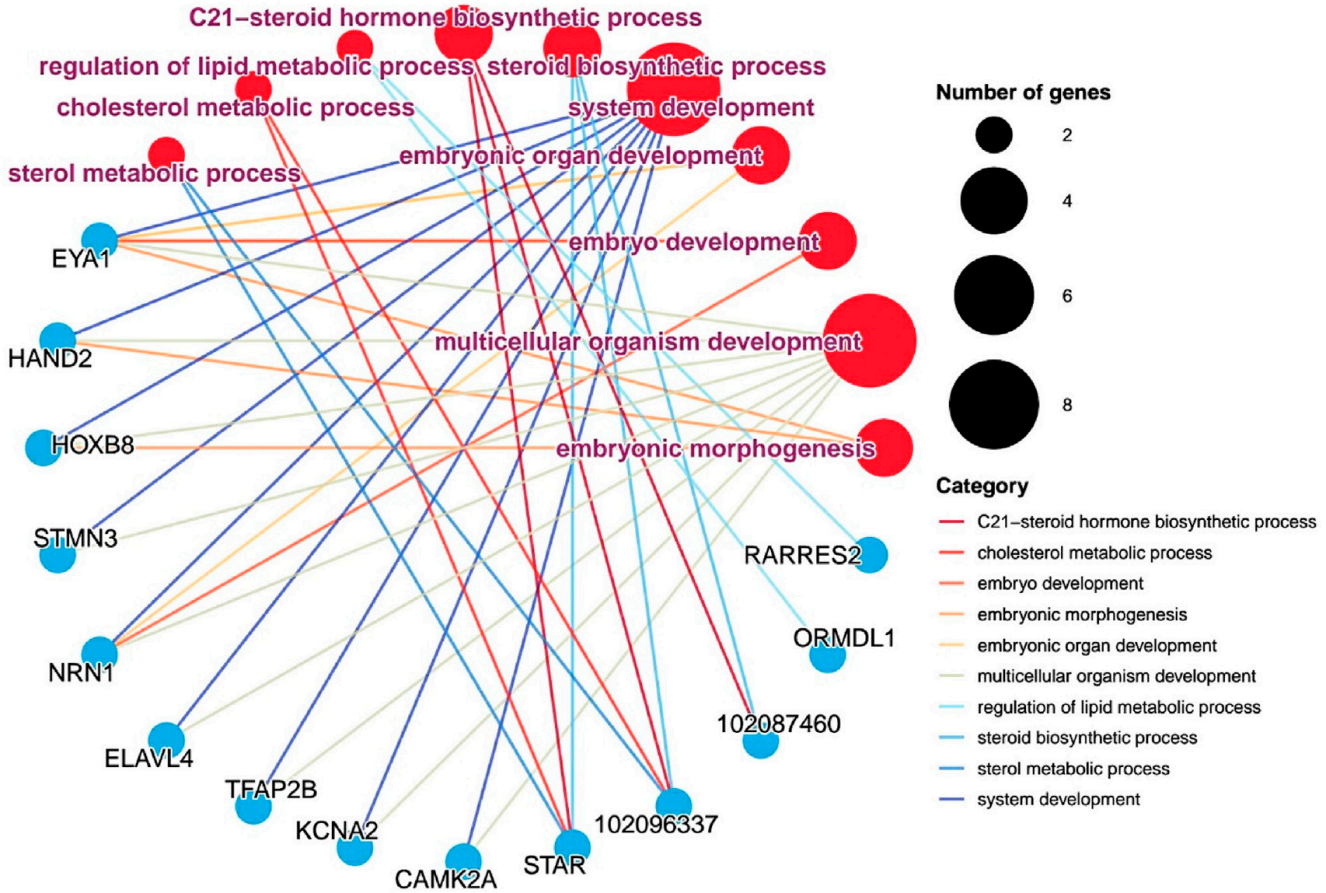
Gene - pathway interaction network analysis. Pathways and genes are represented by circular shapes in different colors. Pathways are represented by red dots, with larger nodes indicating more genes contained in the pathway and smaller nodes indicating fewer genes; meanwhile, pathway names are marked in purple; blue dots represent different genes.

Steroid hormones play a vitally important regulatory role during the egg-laying interval of pigeons. During the egg interval period, steroid hormones are maintained at a relatively high concentration level, which provides a solid guarantee for the normal development of follicles. Based on this, we have sorted out the information on differentially expressed genes and related pathway information that are involved in the processes of steroid hormone production or embryonic development in ovarian tissues, and have drawn a gene-pathway network diagram. The aim is to further uncover the mysteries of pigeon reproductive physiology and lay a solid foundation for improving the reproductive performance of pigeons. As shown in Figure 5, in the ovarian tissue, we identified such as sterol metabolic process, C21-sterol hormone biosynthetic process, cholesterol metabolic process. The steroidogenic acute regulatory protein (StAR) was identified. This key gene plays a crucial regulatory role in the process of steroid hormone synthesis. In addition, we also identified embryonic morphogenesis, system development, embryo development, and organ development (*P* < 0.05), and also identified some genes, such as *EYA1* (eyes absent homolog 1, *EYA1*), *HAND2* (heart and neural crest derivatives-expressed protein 2, *HAND2*), *HOXB8* (homeobox b8, *HOXB8*), *NRN*1 (neuritin 1, *NRN1*), and *ELAVL4* (elav - like rna binding protein 4, *ELAVL4*). These genes can appear in multiple pathways simultaneously, which indicates that in the overall developmental regulatory network system of pigeon ovaries, they are most likely to play extremely crucial and intricate roles, profoundly influencing a series of complex processes in pigeon ovaries from the initial stage of embryonic development to the formation of mature follicles, and thus potentially having a decisive impact on the important reproductive trait of egg-laying interval at the root level.

## 4 Discussion

Pigeon breeding has important economic significance on a global scale. With the continuous increase in people’s demand for high-quality proteins, the pigeon industry continues to develop and grow. However, due to the special reproductive function of pigeons, their egg-laying interval is relatively long, which limits efficient breeding. In fact, different breeds of meat pigeons show significant differences in reproductive performance, but the relevant mechanisms are not clear.

Egg-laying interval is a key indicator of pigeon reproductive performance, and significant breed-specific differences have been reported in previous studies. In the present study, we further validated this trait in European meat pigeons and Shiqi pigeons through a 6-month phenotypic survey. Our phenotypic statistics showed that European meat pigeons had a shorter egg-laying interval (32.76 ± 3.25 days) than Shiqi pigeons (33.11 ± 3.86 days) with significant difference (*P* = 0.024), which is consistent with previous reports on the reproductive characteristics of the two breeds (Chunyu et al., 2018; Tang and Mu, 2018). Although there are slight differences in absolute values, the consistent trend confirms that the egg-laying interval difference is a stable breed-specific trait, providing a reliable basis for subsequent transcriptome analysis of the hypothalamus-pituitary-ovary (HPO) axis.

Laying performance is one of the economically important traits in poultry production. The HPO axis plays a central role in regulating reproductive physiology in animals (Zhao et al., 2023). In pigeons, the HPO axis coordinates a series of key reproductive processes ranging from the initiation of sexual maturation, follicular development, ovulation and hormone secretion (Chunyu et al., 2018). However, current research on the HPO axis in meat pigeons remains relatively limited. Existing studies have confirmed that different pigeon breeds exhibit distinct reproductive performance - some show high fecundity with short inter-laying intervals, while others have lower fecundity, but the molecular mechanisms driving these differences-especially how gene expression patterns, hormone regulatory networks, and signal transduction pathways in the HPO axis shape such phenotypic variations-remain largely unclear. Against this backdrop, our study collected hypothalamus, pituitary, and ovary tissues from European meat pigeons and Shiqi pigeons during the egg-laying interval for transcriptome sequencing, aiming to identify key genes and regulatory networks associated with inter-laying intervals. GO functional analysis revealed that reproduction-related biological processes were differentially enriched across HPO axis tissues: In the hypothalamus, DEGs were explicitly enriched in processes directly involved in reproductive regulation, such as thyroid hormone transport; in the ovary, DEGs were enriched in reproduction-associated developmental processes, such as embryonic morphogenesis, embryonic organ morphogenesis and steroid biosynthetic process-a core pathway for follicular maturation and ovulation. For the pituitary, although no explicitly reproduction-labeled biological processes were detected, the enriched pathways included calcium-mediated signaling, intracellular signal transduction, serve as essential upstream regulatory links for reproductive hormone secretion (Xiao et al., 2002; Stojilkovic et al., 2005; Liu et al., 2006), providing a functional basis for the pituitary’s role as a “signal relay station” in the HPO axis.” Collectively, these tissue-specific enrichment features and their coordinated functional effects collectively reflect an comprehensive regulatory mechanism of the HPO axis in shaping the egg-laying interval of pigeons.

KEGG pathway enrichment analysis found that among the top 20 pathways, the thyroid hormone signaling pathway was enriched in both the hypothalamus and pituitary, and this pathway has been confirmed to be involved in the regulation of the reproductive axis by regulating the synthesis and secretion of hormones such as LH and FSH (Kang et al., 2020; Ren and Zhu, 2022). In the hypothalamus, the balanced expression of the thyroid hormone-activating enzyme gene Dio2 and the inactivating enzyme gene Dio3 is critical for photoperiod-induced gonadal development (Watanabe et al., 2007). Additionally, thyroid hormones (THs) influence reproductive processes through multiple mechanisms: they regulate the secretion and function of key reproductive hormones, interact directly with estrogen, progesterone, FSH, LH, and prolactin to affect ovarian and uterine function, and modulate GnRH release in the HPG axis (Silva et al., 2018; Torre et al., 2020). This role is evolutionarily conserved, as evidenced in mammals-thyroidectomy disrupts seasonal reproductive transitions in sheep, underscoring the thyroid hormone signaling pathway as a phylogenetically conserved regulator of reproduction (Webster et al., 1991).

In ovarian tissue, KEGG analysis of differentially expressed genes revealed that the steroid hormone biosynthesis pathway was significantly enriched, which is closely related to follicular development and ovulation. Steroid hormones are central to follicle development and ovulation, with their synthesis genes exhibiting stage-specific expression patterns across follicular maturation (Johnson, 2014). Progesterone, a key steroid hormone in this pathway, not only drives follicle development but also serves as a precursor for androgen and estrogen synthesis (Zhao et al., 2023); in poultry, it is primarily synthesized by follicular granulosa cells. The biosynthesis process begins with steroidogenic acute regulatory protein (StAR) transporting cholesterol to the inner mitochondrial membrane, where cholesterol side-chain cleavage enzyme (CYP11A1) converts it to pregnenolone - the foundational step in steroidogenesis (Lee et al., 1998; Sechman et al., 2016). Studies have found that with the development and maturation of graded follicles in turkeys, the synthesis of progesterone continuously increases, while the synthesis amounts of testosterone and estradiol continuously decrease (Kristen et al., 2019). Six hours before ovulation, the progesterone level in chicken serum increases. Progesterone can stimulate the formation of the pre-ovulatory LH peak (Etches and Cheng, 1981). Together, these findings suggest that the thyroid hormone signaling pathway (hypothalamic-pituitary) and the steroid hormone biosynthesis pathway (ovary) are the coordinators and regulators of egg-laying intervals. The former controls the secretion of upstream reproductive hormones, while the latter controls the maturation of downstream follicles and the timing of ovulation. This tissue-specific pathway enrichment aligns with the functional division of the HPO axis, providing a molecular framework for understanding breed-specific differences in pigeon egg-laying intervals.

Under the action of cytochrome oxidase, cholesterol is converted into oxysterol within mitochondria. The theca cells and granulosa cells in the ovary utilize it to synthesize reproductive hormones (Douglas et al., 2017). The protein encoded by the StAR gene plays a key role in the acute regulatory stage of steroid hormone synthesis and can promote the conversion of cholesterol into pregnenolone. Its mechanism of action is to mediate the transport of cholesterol from the outer mitochondrial membrane to the inner mitochondrial membrane, and then to cleave cholesterol to generate pregnenolone (Sechman, 2013). Previous studies have shown that StAR exists not only in steroid hormone-producing tissues such as the adrenal gland, testis and ovary, but also is widely distributed in many other tissues, and its expression level is relatively high in the ovary (Reyland et al., 2000). StAR has an indispensable role in the process of ovarian development and ovulation. Additionally, genes related to embryonic and tissue organ development, such as *EYA1*, *HAND2*, *HOXB8* and *NRN1*, merit attention. *EYA1* is not only expressed during somitogenesis but also participates in the morphogenesis of other organs (Raphaelle et al., 2007). *HAND2* belongs to the basic helix-loop-helix (bHLH) family of transcription factors and is expressed in the heart, limb buds and numerous neural crest derivatives during embryogenesis. Mice with *HAND2* gene knockout (HAND2^−/−^) exhibit phenotypes of severe hypoplasia of the right ventricle and growth retardation (Hiroyuki et al., 2001). The *Hoxb8* gene is a member of the homeobox gene family. This family is highly conserved during the evolutionary process and exerts regulatory functions upstream of or within multiple processes such as dorsal spinal cord development and embryonic skeletal system morphogenesis (Wu et al., 2024). Mechanisms of ovarian development and embryonic and tissue organ development, providing a solid theoretical basis and novel research ideas for improving the reproductive performance of pigeons.

## 5 Conclusion

In this study, transcriptome sequencing of hypothalamus, pituitary and ovary tissues from European meat pigeons and Shiqi pigeons identified DEGs. Significant gene expression differences were found in their HPO axis tissues, with the ovary showing the most DEGs (199), indicating it as a key tissue regulating the egg-laying interval. Pathway enrichment analysis revealed that the thyroid hormone signaling pathway (co-enriched in hypothalamus and pituitary) and steroid hormone synthesis pathway (enriched in ovary) are likely core regulatory pathways for the egg-laying interval: the former regulates the laying cycle by controlling reproductive hormone secretion, while the latter influences it by affecting follicle maturation. Key genes including StAR (steroid synthesis-related) and EYA1, HAND2, HOXB8, NRN1 (embryonic and tissue development-related) were identified, which may affect the egg-laying interval by regulating steroid hormone synthesis and follicle development. This study improves understanding of the HPO axis in regulating the egg-laying interval and may help enhance pigeon reproductive efficiency.

## Data Availability

The data of this study is publicly available, with the valid accession number PRJNA1205914. The data is archived in the National Center for Biotechnology Information (NCBI) database, and the link is https://www.ncbi.nlm.nih.gov/.
