# Supplementary material for: Transcriptome profiling of hypothalamus-pituitary-ovary axis provides insights into egg-laying interval differences between european meat pigeons and shiqi pigeons
Source: Front Genet. 2025 Oct 16;16:1676255. doi: 10.3389/fgene.2025.1676255 (PMC12571566; doi:10.3389/fgene.2025.1676255)
Supplement: Supplementary file 1 [file Table1.docx]

Supplementary Material

# Supplementary Tables

Supplementary table 1. Data filtering

| Sample | Total Raw Reads (M) | Total Clean Reads (M) | Total Clean Bases(Gb) | Clean Reads Q20(%) | Clean Reads Q30(%) | Clean Reads Ratio(%) |
| --- | --- | --- | --- | --- | --- | --- |
| EP1 | 43.69 | 42.42 | 6.36 | 98.5 | 94.06 | 97.1 |
| EP2 | 43.69 | 42.81 | 6.42 | 98.62 | 94.53 | 97.98 |
| EP3 | 43.69 | 42.35 | 6.35 | 98.56 | 94.34 | 96.94 |
| EP4 | 43.69 | 42.18 | 6.33 | 98.65 | 94.6 | 96.55 |
| EP5 | 43.69 | 42.5 | 6.37 | 98.69 | 94.79 | 97.26 |
| EO1 | 43.69 | 42.29 | 6.34 | 98.67 | 94.75 | 96.79 |
| EO 2 | 43.69 | 42.54 | 6.38 | 98.73 | 94.95 | 97.37 |
| EO 3 | 43.69 | 42.76 | 6.41 | 98.69 | 94.74 | 97.88 |
| EO 4 | 43.69 | 42.61 | 6.39 | 98.62 | 94.53 | 97.53 |
| EO 5 | 43.69 | 42.66 | 6.4 | 98.72 | 94.97 | 97.65 |
| EH1 | 43.69 | 42.67 | 6.4 | 98.52 | 94.1 | 97.67 |
| EH 2 | 43.69 | 42.66 | 6.4 | 98.59 | 94.44 | 97.65 |
| EH 3 | 43.69 | 42.42 | 6.36 | 98.67 | 94.74 | 97.1 |
| EH 4 | 43.69 | 42.44 | 6.37 | 98.61 | 94.42 | 97.13 |
| EH 5 | 43.69 | 42.82 | 6.42 | 98.65 | 94.63 | 98.01 |
| SP1 | 43.69 | 42.07 | 6.31 | 98.55 | 94.18 | 96.29 |
| SP 2 | 43.69 | 42.62 | 6.39 | 98.73 | 94.96 | 97.54 |
| SP 3 | 43.69 | 42.49 | 6.37 | 98.75 | 95.04 | 97.26 |
| SP 4 | 43.69 | 42.77 | 6.42 | 98.62 | 94.55 | 97.89 |
| SP 5 | 43.69 | 42.51 | 6.38 | 98.68 | 94.72 | 97.29 |
| SO1 | 43.69 | 42.62 | 6.39 | 98.68 | 94.71 | 97.55 |
| SO 2 | 43.69 | 42.61 | 6.39 | 98.69 | 94.78 | 97.52 |
| SO 3 | 43.69 | 42.19 | 6.33 | 98.7 | 94.85 | 96.58 |
| SO 4 | 43.69 | 42.64 | 6.4 | 98.63 | 94.47 | 97.59 |
| SO 5 | 43.69 | 42.61 | 6.39 | 98.77 | 95.14 | 97.53 |
| SH1 | 43.69 | 42.68 | 6.4 | 98.67 | 94.64 | 97.68 |
| SH2 | 43.69 | 42.61 | 6.39 | 98.67 | 94.75 | 97.52 |
| SH3 | 43.69 | 42.2 | 6.33 | 98.69 | 94.8 | 96.58 |
| SH4 | 43.69 | 42.35 | 6.35 | 98.64 | 94.53 | 96.93 |
| EP1 | 43.69 | 42.66 | 6.4 | 98.58 | 94.34 | 97.65 |

Supplementary table 2. Reference genome matching results

| Sample | Total Clean Reads (M) | Total Mapping(%) | Uniquely Mapping(%) |
| --- | --- | --- | --- |
| EP1 | 42.42 | 77.68 | 76.73 |
| EP2 | 42.81 | 58.95 | 58.17 |
| EP3 | 42.35 | 65.92 | 65.1 |
| EP4 | 42.18 | 73.17 | 72.25 |
| EP5 | 42.5 | 77.24 | 76.29 |
| EO1 | 42.29 | 87.79 | 86.38 |
| EO 2 | 42.54 | 88.19 | 86.73 |
| EO 3 | 42.76 | 87.09 | 85.63 |
| EO 4 | 42.61 | 67.55 | 66.57 |
| EO 5 | 42.66 | 73.39 | 72.34 |
| EH1 | 42.67 | 75.19 | 74.15 |
| EH 2 | 42.66 | 61.01 | 60.25 |
| EH 3 | 42.42 | 71.53 | 70.63 |
| EH 4 | 42.44 | 67.18 | 66.38 |
| EH 5 | 42.82 | 74.9 | 73.99 |
| SP1 | 42.07 | 74.53 | 73.66 |
| SP 2 | 42.62 | 82.1 | 81.05 |
| SP 3 | 42.49 | 81.51 | 80.47 |
| SP 4 | 42.77 | 76.31 | 75.34 |
| SP 5 | 42.51 | 74.25 | 73.36 |
| SO1 | 42.62 | 88.65 | 87.3 |
| SO 2 | 42.61 | 90.25 | 88.59 |
| SO 3 | 42.19 | 87.07 | 85.68 |
| SO 4 | 42.64 | 88.37 | 86.99 |
| SO 5 | 42.61 | 88.72 | 87.35 |
| SH1 | 42.68 | 79.43 | 78 |
| SH2 | 42.61 | 70.02 | 69.16 |
| SH3 | 42.2 | 74.81 | 73.81 |
| SH4 | 42.35 | 77.08 | 76.14 |
| SH5 | 42.66 | 79.94 | 78.74 |
